# Commentary on the Issue of *Leishmania* Infection: Focus on Some Pathogenetic, Clinical, and Epidemiological Aspects

**DOI:** 10.3390/vetsci12060536

**Published:** 2025-06-01

**Authors:** Stefania Hanau, Martina Maritati, Carlo Contini, Alessandro Trentini, Maria Cristina Manfrinato, Shawgi Hago Almugadam

**Affiliations:** 1 Department of Neuroscience & Rehabilitation, University of Ferrara, 44124 Ferrara, Italy; 2 Department of Translational Medicine and for Romagna, University of Ferrara, 44124 Ferrara, Italy; mrtmtn@unife.it; 3 Department of Medical Sciences, Infectious Diseases, University of Ferrara, 44124 Ferrara, Italy; cnc@unife.it; 4 Department of Environmental and Prevention Sciences, University of Ferrara, 44124 Ferrara, Italy; trnlsn@unife.it; 5 Department of Chemical, Pharmaceutical and Agricultural Sciences, University of Ferrara, 44124 Ferrara, Italy; mmc@unife.it; 6 Faculty of Medical Laboratory Sciences, University of Khartoum, Nile Avenue, P.O. Box 321, Khartoum 51111, Sudan; shawgi_78@yahoo.com

**Keywords:** *Leishmania*, leishmaniasis, vector-borne disease, neglected tropical disease, immunological status, parasite

## Abstract

Leishmaniasis are infectious diseases caused by several parasitic species of *Leishmania*, mainly transmitted by the bite of infected phlebotomine sandflies. Humans, dogs, rodents, and other domestic and wild animals can act as reservoir hosts for the different *Leishmania* species. It is a neglected tropical disease that is endemic in Asia, the Middle East, North and East Africa, the Mediterranean region, and South and Central America. Clinical manifestations and disease severity depend on the species of the infecting parasites and the immunity status of the host. *Leishmania* represses the protective host immune response by manipulating the macrophage function, subverting cytokine expression to favor its survival and dissemination. A balance between pro-inflammatory and regulatory cells is necessary to bring a positive outcome. Accurate diagnosis and effective treatment represent the cornerstone in the control of this disease, although these are difficult in an environment of precariousness and poverty. Some recent studies highlighted the progressing work on diagnosis and treatments, such as the development of new benzimidazole-triazole derivatives for blocking the parasite growth, feline leishmaniasis with a comparison of immune responses in cats and dogs, and a transglutaminase that has been purified from *L. infantum*. The results of these studies could open new avenues in combating leishmaniasis.

## Simple Summary

Leishmaniasis is a complex of vector-borne, poverty-related diseases that includes visceral, cutaneous, and mucocutaneous forms. It is caused by protist parasites and transmitted by sand fly insects, and its occurrence is strictly dependent on the immunity state of the host. It can be anthroponotic (human to vector to human) in the Indian subcontinent and Asia, while it is mainly zoonotic (animal to vector to human) in Africa, Europe, and the Americas, where dogs and rodents can act as main reservoir hosts. Visceral leishmaniasis infections acquired in the Mediterranean region usually remain asymptomatic in healthy adults but can progress seriously in children and immunocompromised individuals. Climate change, deficiencies in environmental sanitation, and precarious conditions, such as those caused by wars, might be responsible for the infection upsurge and extension of transmission to regions previously regarded as non-endemic. *Leishmania* suppresses the immune function of the host by manipulating macrophage responses and subverting cytokine expression to favor its survival and dissemination. A balance between pro-inflammatory and regulatory cells is necessary to bring a positive outcome. Diagnosis, treatment, and prognosis are fundamental, with a necessity for the development of new, efficacious, safe drugs. Therefore, gaining in-depth knowledge of these parasites with a focus on new potential drug targets is extremely important.

## 1. Introduction and Epidemiological Considerations

A commentary on leishmaniasis is appropriate to the current state of rapid environmental change and disputes in the world. Indeed, wars increase the incidence of infectious diseases through population displacement with the creation of overcrowded regions, a breakdown in healthcare facilities and vital infrastructure, and the vulnerability of the population [1,2]. For vector-borne infectious diseases, the lack or complexity of vector control is an additional factor; this is also the case for leishmaniasis [3].

Leishmaniasis describes a clique of infectious diseases caused by several parasitic species of *Leishmania* that are mainly transmitted by the bite of infected phlebotomine sandflies but also via infected transplants, infected blood, or vertically, from mother to child [4,5,6]. Humans, dogs, rodents, and other domestic and wild animals are reservoirs for the different *Leishmania* species [7]. Cats, horses, lizards, bats, and many other animals can also be infected [8,9]. This neglected tropical disease (NTD) is endemic in Asia, the Middle East, North and East Africa, the Mediterranean region, and South and Central America [10]. Leishmaniasis also occurs in civilians travelling to *Leishmania* foci abroad and in soldiers returning from endemic regions [11]. The prevalence of the disease is higher in poor populations, potentially affecting more than one billion people worldwide [12]. Due to climate change and the extending wars, poverty and migration from endemic areas are growing, and epidemiological surveillance is lacking in many regions [13]. Rural areas, deforestation, and deficiencies in environmental sanitation are significant risk factors for *Leishmania* transmission [14]. Moreover, global warming is causing both geographical expansion of the insect vector and prolongation of its seasonal activity [15].

Clinical manifestations and disease severity depend on the infecting parasite species, the type of insect vector with its inoculated saliva, the presence of virus within the parasite itself, and the immune status of the host [8,16,17]. In canids, severe leishmaniasis manifests in a viscerocutaneous form (CVL), while in humans, there are three main forms: visceral leishmaniasis (VL), cutaneous leishmaniasis (CL), and mucocutaneous leishmaniasis (MCL) [7,10,18]. VL, also known as kala-azar and usually caused by *L. donovani* and *L. infantum* (also called *L. chagasi*), is almost always fatal without treatment. CL, caused by *L. tropica*, *L. major*, *L. aethiopica*, *L. infantum*, and *L. donovani* in the Old World and by species from the *L. mexicana* complex or from the *Viannia* subgenus, as well as *L. infantum/L. chagasi*, in the New World, is the most common form of leishmaniasis [7,19,20,21]. MCL is caused mainly by *L. braziliensis*, *L. panamensis* (both belonging to the *Viannia* subgenus), and *L. aethiopica*, but also other species in either different regions or in immunocompromised individuals, elderly people, or people with delayed healing of CL lesions. It leads to partial or total destruction of mucous membranes of the nose, mouth, and throat, with over 90% of cases occurring in Bolivia, Ethiopia, Brazil, and Peru [7,20,21]. Post-kala-azar dermal leishmaniasis (PKDL) is a cutaneous and sometimes mucosal manifestation, prevalent in East Africa and South Asia, that occurs after (even many years after remission) or during treatment of VL caused by *L. donovani*; however, it can also occur in HIV–*L. infantum* co-infections [12,20,22].

There are excellent reviews on leishmaniasis in the scientific literature, covering various aspects of the disease and its different forms, only a few of which are cited in this commentary, which points to shed light once again on these complex and neglected parasitic infections (Refs. [18,19,20,21,22,23,24,25,26,27,28,29] and more). The special issue of *Veterinary Sciences* on “*Leishmania* Infection: Pathogenesis, Clinical and Epidemiological Aspects”, of which our first author was the guest editor, recently published articles on different facets, including the following: in vitro cultivation of *Leishmania* and *Trypanosoma* parasites [30]; a new diagnostic test useful for both humans and animals based on the recombinant chimeric protein Q5 [31]; a transglutaminase purified from *L. infantum* (*Li*) [32]; feline leishmaniasis (FeL) [33]; a comparison of immune responses in cats and dogs [34]; studies on the clinical effects of domperidone on dogs [35]; and in vitro antileishmanial effects of new benzimidazole-triazole derivatives [36].

## 2. Pathogenesis and Immune Traits

CD4^+^ T cells and T helper type 1 (Th1) cellular immune responses characterized by the production of the proinflammatory cytokines interferon γ (IFN-γ) and Tumor Necrosis Factor α (TNF-α) are critical for controlling *Leishmania* and providing lifelong immunity against reinfection [19,21,25]. Th17 with IL17 and other secreted cytokines, such as IL2, are also associated with protection against visceral leishmaniasis [37]. However, excessive levels of cytokines, even proinflammatory ones, cause severe forms, such as the human CL and MCL [25]. The parasite invades and multiplies in cells of the myeloid system inside parasitophorous vacuoles, and it is able to contrast production of nitric oxide, the reactive nitrogen form necessary for parasite clearance by phagocytes [38]. One of the *Leishmania*’s strategies to disrupt macrophage activation signaling is to inhibit autophagy by activating the phosphoinositide 3-kinase/protein kinase B (PI3K/AKT) pathway [39]. IL10, together with Transforming Growth Factor β (TGF-β), is typical for a Th2 cellular immune response and supports the progression of leishmaniasis with persistent lesions and dissemination to other tissues, including bone marrow, lymph nodes, spleen, and liver. From a mechanistic point of view, it has been shown to inhibit autophagy through AKT activation [40]. At the same time, the modulation of cytokine expression depends on the binding between specific *Leishmania* virulence factors and immune cell receptors [39]. These receptors are Toll-like receptors (TLRs), which also recognize microbial components in the early phase of infection [41].

Following parasite inoculation into the dermis of a vertebrate host, complement activation and neutrophil action are among the first responses. Many types of resident cells are involved even earlier, such as damaged tissue with chemotactic signals, dermal macrophages, Langerhans dendritic cells (DCs), fibroblasts, mast cells, and keratinocytes. Many of these cells engulf the *Leishmania* metacyclic promastigotes and/or produce chemokines and cytokines that, together with the processing and presentation of antigens by DCs and macrophages, allow for T cell activation [42,43]. IL12 secreted from antigen-presenting cells (APCs) is essential for the polarization of naïve T cells toward a Th1 subset, as well as monocytes and natural killer (NK) cells, the latter producing pro- and anti-inflammatory cytokines, growth factors, and chemokines [42]. Immunoglobulins are also produced against *Leishmania*, which are specific and effective in the early stages of the disease but non-specific in chronic infection, leading to the deposition of immune complexes, for example, in the kidney in VL [17,19,44,45,46,47,48,49,50]. Renal damage often causes death among dogs with canine leishmaniasis (CanL) [45,46]. T cells and B cells interact during CanL, with B cells acting as additional antigen-presenting cells (APCs), activating T helper cells [45]. As a result of prolonged antigen exposure, T cell exhaustion with associated decrease in IFN-γ and increase in IL10, also produced by regulatory B cells, occurs [51]. Both innate and adaptive immunity are involved in the disease control or progression, and a balance between pro-inflammatory and regulatory T cells is necessary to bring a positive outcome [19]. In *L. major* models of chronic infection, IL7R-expressing central memory T cells and Th1 effector cells contribute to concomitant immunity [45,52,53].

*Leishmania* suppresses the protective immune activation of the host by manipulating macrophage function and subverting cytokine expression to favor its survival and dissemination. A shift to an anti-inflammatory (macrophage M2/Th2) milieu promotes parasite persistence [54]. The gene expression pattern has been shown to be dysregulated in poverty for the endosome organization pathway, which is critical for immune cells [55]. Immunosuppressive effects are caused by stress conditions as well as are present in immunosuppressed patients, in those with AIDS with less than 50 CD4+/µL, in malignant or immunological disorders under chemotherapy, biotechnological drugs (BTs), corticosteroids, radiation therapy, in severe rheumatic diseases, and in hematopoietic stem cell and solid organ transplantation [19,56,57]. All of these conditions increase susceptibility to *Leishmania* infection. Also, a reinfection and/or a reactivation of latent *Leishmania*, often in different organs, like skin in PKDL, are possible [22,58]. While in the majority of cases, East African PKDL heals spontaneously, the South Asian variant requires prompt diagnosis and treatment [7,59]. This variant of PKDL is a chronic form, and a CD8+ T cell increase in dermal inflammatory infiltrate is one of its features [54,60,61]. Genetic variants of *Leishmania* show diverse tissue tropism, giving rise to atypical infections, such as atypical cutaneous leishmaniasis (ACL), caused by *L. donovani* in Southeast Asian endemic regions [62]. Gene polymorphisms, related to genes encoding immunological mediators, receptors, and HLA class II histocompatibility antigens, are known to confer some resistance to leishmaniasis, while others seem to confer susceptibility, such as the IFN-γ receptor polymorphism, which decreases responsiveness to IFN-γ in PKDL [26,54,60,63].

## 3. Clinical Aspects

Among the main clinical signs of CanL caused by *L. infantum* in susceptible dogs are lymph node enlargement, weight loss, exfoliative dermatitis, onychogryphosis, alopecia, ulcers, and ocular alterations [64]. In humans, VL usually remains asymptomatic in healthy adults but can progress seriously in children and immunocompromised individuals. It causes fever, malaise, weight loss, possibly splenomegaly, hepatomegaly, anemia, leukopenia, thrombocytopenia, hyperglobulinemia, and other severe complications as the disease progresses. In East Africa, lymphadenopathy may be the only clinical manifestation [7,20,54]. For CL, a wide variety of skin lesions are reported, mainly ulcers, on exposed parts of the body, with possible secondary infections often caused by staphylococcal species [2]. Single nodules or ulcers with *L. infantum* and multiple ones with *L. tropica* and *L. major* can heal spontaneously in months or within 1 year and may leave disfiguring scars. *L. aethiopica* gives mainly nodular lesions or, less frequently, diffuse cutaneous leishmaniasis that do not heal spontaneously. Mucosal lesions in the mouth or larynx or lesions in the nose or on the genital mucosa by all the species are possible in elderly or immunosuppressed people. The American species cause localized (spontaneously healing in some months), disseminated, diffuse, and atypical CL and severe MCL (also known as espundia) that do not heal spontaneously. For the sequelae of leishmaniasis, CL recidivans are the lupoid or tubercoloid chronic forms, while one of the features of PKDL is hypopigmentation [7,12,59].

## 4. Prevention

Accurate diagnosis and effective treatment represent the cornerstone in the control of this disease, although these are difficult to achieve in an environment of precariousness and poverty. In the Indian subcontinent, liposomal amphotericin B (L-AmB) has been available free of charge in public health facilities for more than 10 years, thanks to the World Health Organization and a donation from the manufacturer [27]. In fact, transmission of VL is anthroponotic in South Asia; however, transmission of VL and CL, caused by *L. tropica*, are mixed, zoonotic, and anthroponotic in East Africa. Thus, treating patients promptly and effectively, as well as improved housing, environmental sanitation, and insecticide-based vector control tools, are the actions needed to stop transmission cycles [7,12,65]. Human vaccines remain a global public health priority, and some of the human clinical trials (third-generation vaccines made of DNA antigens) are being tested for prevention, safety, and therapy [28,66,67,68]. Live attenuated parasites (first-generation vaccines) protective effects are assayed in preclinical testing against several *Leishmania* species causing different forms of leishmaniasis [68]. A CRISPR-based approach might help to develop parasite vaccines.

The control of leishmaniasis is also possible by reducing the circulation of the parasite in animals, such as dogs, for instance. We hope that the current third-generation and second-generation canine vaccines (with protein antigens) can not only reduce the severe incidence of the disease in dogs but also reduce the incidence of visceral leishmaniasis in humans, since dogs are the main reservoir hosts for *L. infantum* in the Mediterranean, Central Asia, and Latin America [7,69]. Vaccination may, thus, have both a preventive and a therapeutic role and contribute to lowering the transmission risk to humans [28]. Prevention by the use of repellent collars or other topical formulations has been shown to be useful [70,71]. Vector control insecticide use in the Asia–Pacific Region for the control of leishmaniasis consists mostly of pyrethroids and organochlorines, as reported in 2021, but improvement of the surveillance, insecticide resistance monitoring, and strengthening of entomological expertise are needed. In Iran and Iraq, another reported intervention type is rodent control [72]. In several countries, the elimination of infected dogs is carried out, although many studies report that this practice is neither ethical nor effective in eliminating leishmaniasis [69].

## 5. Diagnosis

After the clinical suspicion of VL or CanL, *Leishmania*-specific laboratory tests are required to confirm diagnosis. The microscopic visualization of *Leishmania* amastigotes (Leishman bodies) on stained smears prepared from aspirates of bone marrow, lymph nodes, or spleen of VL suspects or from materials obtained by scraping, fine-needle aspiration, or biopsy of lesions of CL suspects represents the gold standard method. In severely immunocompromised patients, amastigotes can be demonstrated on smears prepared from buffy coats of peripheral blood. A parasite culture can increase the diagnosis sensitivity but requires a longer time (weeks). Castelli et al. [30] showed that the simple liquid medium RPMI-PY can be efficiently used to grow and study *Trypanosoma cruzi* and some *Leishmania* species, such as the Old World *L. major* and *L. tropica* and the New World *L. amazonensis.* They compared it with the conventional Evans’s modified Tobie (EMTM) and Novy–MacNeal–Nicolle (NNN) biphasic media, used for *Leishmania* and *Trypanosoma*, respectively. The EMTM and NNN media require longer preparation times and need fresh, defibrinated rabbit blood, which is complex to procure. By contrast, *L. braziliensis*, compared to other *Leishmania* species, showed exponential growth only in EMTM. The preference of EMTM is most probably due to a parasite requirement for higher folate concentrations found in this medium.

MALDI-TOF mass spectrometry is used by advanced laboratories to identify *Leishmania* spp. [23], although molecular diagnosis by PCR is the most used and sensitive method that can give a species-specific diagnosis and detect asymptomatic infections. It is, therefore, indicated for MCL diagnosis since the causative parasites are difficult to isolate. Selected sequences exploited as PCR targets include the small subunit rRNA gene (SSrRNA), internal transcribed spacer (ITS), kinetoplast DNA (kDNA), or other highly repetitive genomic DNA sequences [57,64]. Unfortunately, PCR is not feasible in the majority of the *Leishmania* endemic areas, as it requires sophisticated equipment and experienced laboratory personnel. A direct agglutination test (DAT) and immunochromatographic tests (ICTs) based on antigen detection have been developed and can also be used using urine specimens [23]. For the serological diagnosis, DAT, an indirect fluorescent antibody test (IFAT), an enzyme-linked immunosorbent assay (ELISA), and Western blot (WB) analysis can be used to detect antileishmanial antibodies [7,23,73]. Acquired immunity can also be determined using the leishmanin skin test (also called Montenegro’s intradermal test). Rapid ICT tests are greatly used in poor settings. Ferreira de Araujo Paz et al. [31] illustrate the diagnostic potential of a new enzyme-linked immunosorbent assay (ELISA) based on the Q5 chimeric protein, made of three *Li* antigens: Lci2, good for the diagnosis of human VL, and Lci3 and Lci12 antigens, efficient for the diagnosis of CanL [74]. This assay showed high sensitivity and good specificity since many sera from asymptomatic dogs or those with no clinical information available were negative, while the same sera were positive with the rapid immunochromatographic DPP (Dual Path Platform) test. The latter, like Q5, is based on a chimera obtained by fusing multiple tandem repeat sequences of *Li* antigens [75]. A fragment of the motor protein kinesin of the cortical cytoskeleton is shared by the two chimeric proteins (Lci2 in Q5). Lci3 is a hypothetical protein with repetitive motifs, an orthologue of a flagellar attachment zone protein in trypanosomes, while Lci12 is an orthologue of a membrane-associated protein, which is probably involved in vesicular transport [76,77]. The other antigen in DPP is an immunogenic K26 protein, which is predicted to be extracellular and reported to be highly similar to the hydrophilic acylated surface protein B1 (HASPB1). Both K26 and B1 have a 14 amino acid repeat region (11 copies in K26 and at least 22 in HASPB1) [78,79].

Priolo et al. [34] analyzed 109 stray cats and 59 rescued dogs of the CanL endemic area of Cordoba, Spain, for their exposure to *Li* by detecting parasite DNA by PCR, measuring antibodies by ELISA and immunofluorescence antibody tests (IFATs), and, in half of these animals, the specific IFN-γ release upon *Li* soluble antigen (LSA) stimulation of the immune system. In both of the mammalian groups, about one-third of the specimens tested positive for at least one diagnostic test, suggesting that cats and dogs may equally contribute to the endemicity of infection. No agreement between IFAT and ELISA was detected in cats, and no correlation between PCR results and serological tests in both dogs and cats was found. It is not uncommon for PCR results and serological tests to give contrasting results, with positivity in one test not correlating with the other. This is what we found in a previous study on a small number (*n* = 50) of human blood samples from our Italian region (Emilia Romagna), where PCR results and serological test positivity did not correlate (unpublished observation [57]). Blood smears, performed by Priolo et al. [34], on half of the animals were all negative for *Li* amastigotes and hemoparasites, although more than twice as many dogs as cats showed clinical signs compatible with leishmaniasis. However, the prevalence of PCR positivity was similar in the two mammal groups, and this was consistent with their health status and the presence of clinical signs of leishmaniasis, whereas the opposite was true for antibody positivity in both dogs and cats. The authors confirmed that a strong humoral immune response and high blood parasitemia are associated with a lack of cell-specific anti-*Li* IFN-γ production. Finally, like dogs, cats can mount a specific anti-*Leishmania* humoral and cell-mediated immune response but with limited intensity.

## 6. Treatment

Simple CL lesions not associated with the risk of development of MCL can be treated with cryotherapy, thermotherapy, laser therapy, topical paromomycin, and intralesional injections of antimonials. For complex CL, MCL, and VL, the systemic treatments include intravenous administration of L-AmB, intramuscular or intravenous administration of pentavalent antimonials, oral miltefosine and fluconazole, and intramuscular or intravenous administration of pentamidine. Also, for *L. donovani*, intramuscular paramomycin alone or in combination with antimonials is used in East Africa, while combinations of L-AmB with miltefosine or miltefosine with paramomycin are used in South Asia. Thus, therapy is different for each form due to the development of drug resistance, mainly in anthroponotic foci. Moreover, the patient history and immunological status must be considered. In fact, several of these drugs present severe side effects; hence, for instance, in pregnancy, miltefosine cannot be used, or in co-infections with HIV, antimonials are just a last choice given their toxicity. Allopurinol is used alone for CanL or in combination with antimonials to treat leishmaniasis relapses caused by *L. tropica* [7,20,21,23]. Napoli et al. [33] present one of the few clinical and diagnostic reports on FeL, along with data on a 28-month follow-up period. A 13-year-old cat living in an Italian area, considered endemic for CanL and co-infected with FIV, presented with dermatological lesions localized in the eyelid and periocular region, which are not so common. Long-term oral allopurinol administration resulted in a fast resolution and long survival period, although a sudden relapse occurred when therapy was discontinued.

An immunotherapeutic approach was tried, even in combination with conventional treatments, for instance, IFN-γ with antimonials [24]. The use of domperidone as an immunomodulatory drug is widespread in the treatment and prevention of CanL [35,80]. In humans, domperidone is used to treat gastrointestinal disorders and to stimulate prolactin secretion, which is associated with immunostimulatory effects. Donato et al. [35] carried out a pilot study on 17 dogs to investigate a possible pro-arrhythmic side effect of domperidone, as seen in humans. They tested whether there was a delay in cardiac repolarization, seen as a prolonged QT interval in the electrocardiogram. After four weeks of treatment, they found a slight prolongation of the QT interval (corrected for heart rate) in 75% of dogs, although none showed arrhythmias or other cardiac side effects. This preliminary study suggests that a clinical assessment of the dog’s cardiac, electrolyte, and hepatic status should be carried out before administering the drug. Attention should also be paid to other drugs being administered that have been reported to prolong the QT interval, such as antihistamines and antimicrobials or those that inhibit the CYP3A4 detoxification system, such as ketoconazole, itraconazole, erythromycin, and fluoroquinolones.

## 7. Research Avenues

Long treatment regimens are required with current drugs, with most of them having difficult administration ways and high cost, toxicity, and resistance [29,81]. New oral medicines should be developed; hence, nanotechnology-based drug delivery systems, such as that for pentamidine, have been tested in experimental models to increase intestinal absorption and decrease toxicity [24,82]. Research is active in finding the leishmanicidal effects of existing drugs used in other fields and of immunomodulators. Thus, the anti-malarial medications artemisinins, chloroquine, and its derivatives were demonstrated to be active against *Leishmania*. An oxazine derivative of nitroimidazo-oxazole (DNDI-0690) has completed clinical trials for VL and CL [83]. The targets of these compounds are the mitochondrion electron transport complexes, where they cause loss of mitochondrial membrane potential and ATP depletion and a subsequent rapid increase in toxic reactive oxygen species (ROS) that ultimately results in parasite death [84]. These drugs were discovered after exploiting plants used in traditional medicine, and then much work has been performed to find the action mechanism of many vegetal leishmanicidal substances [24,85]. Verbascoside, for instance, a glycoside present in several plants, has been shown to inhibit the parasite arginase, which produces L-ornithine, involved in polyamine synthesis [86]. Polyamines are essential for parasite growth and the synthesis of the specific Trypanosomatidae antioxidant trypanothione [87]. Trypanothione reductase is inhibited by clerodane diterpenes isolated from the Euphorbiacea *Croton cajucara* [88]. Due to their peculiarity and indispensability in tryposonomatids, trypanothione as well as glycolytic pathways are among the classical validated targets for rational drug design. Lead compounds, strongly inhibiting in a selective way these and other proposed targets, have been developed [89,90,91,92]. Eser and Cavus [36] synthesized some new benzimidazole-triazole derivatives that are able to block *L. tropica* growth at concentrations of around 0.24–0.46 mg/mL. Docking shows that a possible target of these compounds is sterol 14-alpha-demethylase, which is reported to be important for *Li* metacyclogenesis (the differentiation of the procyclic promastigotes into the infective metacyclic forms). Inhibitors of this demethylase are already effective antifungal medications.

Gaining an in-depth knowledge of these parasites with a focus on new potential drug targets is extremely important [81]. Almugadam et al. [32] have shown that *Li* promastigotes possess a Ca^2+^- and GTP-dependent transglutaminase (TGase), with an apparent mass of 54 kDa, which is similar to the molecular weight of the TGase of the parasite *Brugia malayi*. It displayed cross-reactivity with polyclonal antibodies produced against a conserved region of the catalytic core of human TGase 2. This enzyme is associated with cell death and is important in *Leishmania* infectivity to both sand fly vectors and mammalian hosts. Brobey and Song [93] showed that the inhibition of a similar transamidating enzyme suppresses the growth of *L. amazonensis* and that among the TGase substrates is GP63, leishmaniolysin, which is essential for *Leishmania*’s immunosuppressive and pro-parasitic action. In fact, this zinc-metalloprotease inactivates the complement factor C3b, hampering the formation of the membrane attack complex (MAC), which usually causes a pore on the surface of the pathogen. The binding of the inactive iC3b to specific CR receptors allows for parasite phagocytosis by macrophages with no induction of oxidative bursts [94,95]. Furthermore, this glycoprotein is important for the *Leishmania* amastigote stage survival inside the macrophage phagolysosomes and subversion of signaling above all in these cells [96].

## 8. Conclusions

According to WHO, more than 1 billion people live in areas endemic for leishmaniasis and are at risk of infection. An estimated 30,000 new cases of VL and more than 1 million new cases of CL occur annually. In the WHO road map for 2021–2030, critical actions required for VL are as follows: enabling early case detection to ensure prompt treatment, ensuring the supply of medicines, especially for children and young adults who make up 50–70% of the affected population, and developing more effective and user-friendly treatments and diagnostics, especially for East Africa. For CL, besides developing cheap and easy-to-administer oral or topical treatments, improving the sensitivity of rapid diagnostic tests, increasing surveillance, and establishing a patient database for the monitoring of the impact of interventions are other critical actions. Further challenges are as follows: (1) diagnosis in patients with acquired immunodeficiency, presenting atypical VL and diffuse CL, usually resistant to treatment; (2) diagnosis in non-endemic regions, especially for CL, is sometimes misdiagnosed with skin cancer or other conditions, thereby delaying treatment [2,7,12,65].

A recent meta-analysis on CanL caused by *L. infantum* estimates the global prevalence of leishmaniasis in dogs as 15.2% [71]. In America, *L. braziliensis* is also a widespread agent of CanL [97]. Prevention and control of *Leishmania* infection in dogs is important as well as for other pets, like domestic cats, which could act as reservoir hosts. Anyway, outbreaks showed that wild animals living in recently urbanized lands or new parks in the city outskirts (like hares) can play a role as active reservoirs [98,99]. Surveillance and reports are important; in fact, climate change is causing the extension of leishmaniasis to new regions, as reported, for instance, in Europe, North Africa, and Iran [69,100].

A good immune status is essential to win against the *Leishmania* attack, as the disease victims are the poorest. It is more important to ensure a dignified life in every part of the world, while donating money for developing safe and efficient drugs, rapid diagnostic tests, and monitoring NTD can only be the second line. The knowledge of the parasite cycle and of the biology of these ancient parasites is indispensable.

## Data Availability

The data presented in this study are available on request from the corresponding author.
